# Physicochemical characterisation of reduced graphene oxide for conductive thin films†‡

**DOI:** 10.1039/c8ra08849g

**Published:** 2018-11-07

**Authors:** Elizabeth J. Legge, Muhammad Ahmad, Christopher T. G. Smith, Barry Brennan, Christopher A. Mills, Vlad Stolojan, Andrew J. Pollard, S. Ravi P. Silva

**Affiliations:** Advanced Technology Institute, University of Surrey Guildford Surrey GU2 7XH UK s.silva@surrey.ac.uk; National Physical Laboratory Hampton Road, Teddington, Middlesex TW11 0LW UK; Metallic and Functional Coatings Group, Surface Engineering Department, Tata Steel, Steel and Metals Institute, Swansea University Swansea SA2 8PP UK

## Abstract

Graphene is a desirable material for next generation technology. However, producing high yields of single-layer flakes with industrially applicable methods is currently limited. We introduce a combined process for the reduction of graphene oxide (GO) *via* vitamin C (ascorbic acid) and thermal annealing at temperatures of <150 °C for times of <10 minutes, resulting in electrically conducting thin films with sheet resistances reducing by 8 orders of magnitude to as low as ∼1.3 kΩ □^−1^, suitable for microelectronics, display technology and optoelectronic applications. The in-depth physicochemical characterisation of the products at different stages of GO preparation and reduction allows for further understanding of the process and demonstrates the suitability for industrial production methodologies due to an environmentally-friendly reducing agent, solution processability and no requirement for high temperatures. The presence of the vitamin C lowers the temperature required to thermally reduce the GO into an electrically conducting thin film, making the technique suitable for thermally sensitive substrates, such as low melting point polymers. Simultaneous spray coating and reduction of GO allows for large area deposition of conductive coatings without sacrificing solution processability, often lost through particle agglomeration, making it compatible with industrial processes, and applicable to, for example, the production of sensors, energy devices and flexible conductive electrodes for touchscreens.

## Abstract 2

We introduce a combined process for the reduction of graphene oxide (GO) *via* vitamin C (ascorbic acid) and thermal annealing.

## Graphical abstract

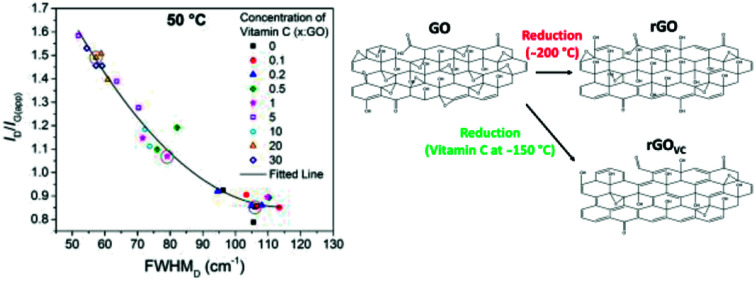

## Introduction

Graphene is a highly desirable material for use in next generation electronics applications due to its optical transparency,^1,2^ high tensile strength,^3^ flexibility^2,4^ and electrical conductivity.^5^ To date however, there are very few industrially applicable methods of manufacture to mass produce high yields of single-layer graphene flakes. Three key graphene production techniques are chemical vapour deposition (CVD),^6–9^ liquid phase exfoliation (LPE)^10–12^ and chemical exfoliation.^13–15^ CVD-grown graphene typically has minimal structural defects,^6^ however high growth temperatures (of up to 1000 °C) are required, as are specific growth substrates. Furthermore, CVD-grown graphene is not suitable for many applications, such as composites and inks. On the other hand, both LPE and chemical exfoliation of graphite produce graphene/graphite flakes at lower temperatures that can be used as an additive and produced on the tonne-scale. LPE produces graphene flakes with few defects,^10^ although the yield of single-layer graphene is low, and these flakes have small lateral sizes of typically <1 μm.^10^ Chemical exfoliation produces a higher yield of single-layer flakes with larger lateral sizes but leads to graphene oxide (GO) or reduced graphene oxide (rGO) flakes with defects (ESI Fig. S1†).^16^

GO, a chemically modified derivative of graphene, is a material with the potential for industrial scale-up,^17–20^ in particular for application in the microelectronics, display and optoelectronics fields. Unlike pristine graphene, GO can be stable in an environmentally friendly dispersion and can be easily deposited as a thin film with little agglomeration, features lacking in its more well-known pure carbon analogue, graphene, but are desirable for industrial processes. Currently, thin films of GO lack some important features, such as the high electrical conductivity found in graphene, but moderate conductivity can be achieved^21–23^ and is suitable for certain applications.^24–27^ One potential use, demonstrated in this work, is for a touch-sensitive capacitive sensor, suitable for use in large area applications such as interactive surfaces.

This work explores the suitability of GO for the production of thin, electrically-conducting coatings. GO is produced from graphite oxide, itself a product of graphite oxidation using the Hummers and Offeman method.^28^ During the chemical process, oxygen functional groups bind to the graphite sheets, weakening the van der Waals forces between the graphene layers. When re-dispersing graphite oxide in water, ultra-sonication of the dispersion produces a high yield of GO flakes.^29^ However, due to the presence of the oxygen functional groups bound to the basal plane the electrical conductivity is reduced.

To be sufficiently electrically conducting the GO must therefore be reduced, typically reaching 10^3^–10^7^ Ω □^−1^.^2,21,30^ Both chemical and thermal reduction techniques remove oxygen functional groups bound to the flakes.^31^ Chemical reduction historically involved the use of hydrazine,^14^ however ascorbic acid (vitamin C) is non-toxic and can produce similar results.^32,33^ Thermal annealing typically requires temperatures of 200 °C or greater in an inert atmosphere but to be industrially relevant reduction at atmospheric conditions and lower temperatures is desirable, and is demonstrated herein. To produce a uniform thin film of GO across a substrate, commercially-scalable spray coating is a suitable method of deposition,^34^ requiring relatively small capital investment. Here, we apply our combined chemical and thermal reduction method to produce rGO and demonstrate the production of large area, spray-coated, thin films, with electrical conductivities of ∼1.3 kΩ □^−1^, competitive with existing methods of rGO production.^1,2,21,35^

When spray coating, uniform reproducible deposition is key to reaching a percolating path across the substrate. Regardless of the electrical conductivity of an individual flake, a pathway of interconnecting flakes must be produced to allow electrical conduction. If optical transparency is a requirement, such as for transparent touchscreens, this path should also be formed with the minimum film thickness possible, as such the spray coating process requires optimisation, not only for spray volume and duration, but also for feedstock solution composition. In this work, by using a combination of isopropanol (IPA) and deionised water as the solvent, the GO dispersion dries soon after contacting the substrate, allowing film deposition at room temperature.

## Experimental

GO was prepared from graphite using the modified Hummers and Offeman process,^28^ as described in the ESI (Section 1.2).† The graphite oxide powder produced from the Hummers and Offeman process was then dispersed in deionised water (2 mg ml^−1^ of GO in H_2_O) and an ultrasonic probe was used to produce an exfoliated GO dispersion. For this sonication step, an ultrasonic processor (Cole Parmer, 750 W) was used for a total sonication time of 10 minutes, consisting of two 10 minute runs with bursts of 5 seconds on and 5 seconds off. Vitamin C was added to the GO solution at a range of concentrations, from 0.2 mg ml^−1^ to 60 mg ml^−1^. Isopropanol (IPA) was then added to the dispersion at a ratio of 1 : 1 with deionised H_2_O. This alcohol co-solvent was used to reduce the agglomeration of the GO flakes, while also aiding the drying process when depositing. The GO dispersion was immediately deposited after adding vitamin C and IPA, unless otherwise stated.

To deposit a thin film of GO, substrates were spray-coated using a gravity-feed air brush (AB931 air brush kit, Sealey) and a nitrogen carrier gas (30 mbar), at a spray rate of approx. 1 ml min^−1^, from a distance of 10–15 cm. To support the sheet resistance measurements and understand the relative film thickness, the transmittance was measured with ultraviolet visible spectroscopy (UV-vis) using a Varian Cary 5000 UV-vis-NIR spectrophotometer. Immediately after spray coating, the substrates were heated at temperatures in the range of 50 °C to 250 °C for times between 0–10 minutes as stated in the Results section.

Raman spectroscopy (inVia, Renishaw Ltd, UK) was performed using a laser of wavelength 514 nm (2.41 eV), with a 100× (0.90 NA) objective lens and a total laser power of less than 405 μW incident on the sample, to determine the level of disorder within the rGO flakes. The samples for Raman spectroscopy were prepared by spray coating silicon substrates and heating for 10 minutes, at the temperatures stated in the results. The D-peak (∼1350 cm^−1^) of the rGO was fitted with a Lorentzian peak, consistent with the literature.^36^ The intensity (height) and full width at half maximum (FWHM) of the D-peak were then obtained from this fitted peak data. For the G-peak (∼1580–1600 cm^−1^) intensity, the apparent peak height (*I*_G(app)_) was taken to allow comparison with other data, since different methods of fitting the G-peak for GO can greatly alter the *I*_D_/*I*_G_ peak intensity ratio.^36–39^

Thermogravimetric Analysis (TGA), was undertaken in air (Q500 TGA, TA Instruments, USA. Balance gas, nitrogen: 40 cm^3^ min^−1^, sample gas, air: 60 cm^3^ min^−1^) to determine the temperature at which reduction of GO and decomposition of vitamin C occurred, as well as identify the level of oxygen functionalisation of the GO or rGO flakes. GO powder and vitamin C were placed on separate platinum crucibles and heated at a rate of 10 °C min^−1^ from room temperature to 800 °C. For TGA of graphene oxide reduced with vitamin C (rGO_VC_), the dispersion was prepared by drop casting rGO_VC_ onto silicon. The sample was heated to 50 °C for 10 minutes and subsequently washed with deionised water to remove vitamin C, after which the sample was dried in a desiccator. Another sample using a polytetrafluoroethylene (PTFE) substrate was also prepared in an identical manner but without the heating or washing steps, to provide a comparison. The dried material was removed from the two substrates and placed in separate platinum crucibles, to be heated under the same conditions as the vitamin C and GO powder.

To measure the electrical conductivity of the spray-coated GO films on glass slides, wire connections were made to the films using Al tape and Ag paste. The wires were connected to a Keithley 4200-SCS analyser for electrical current measurements as a function of time, while the temperature of each sample was increased from room temperature to 250 °C using a hot plate. The temperature was measured using a k-type thermocouple and recorded in real time using a Picotech TC-08 data recorder.

The sheet resistances of the spray-coated GO films were measured using a 4-point collinear probe connected to a Keithley 4200-SCS analyser. The same samples were analysed with UV-vis (Varian Cary 5000 UV-vis-NIR spectrophotometer) to measure the transmittance.

Samples for X-ray photoelectron spectroscopy (XPS) were prepared by drop casting the rGO_VC_ dispersions onto Si wafers so as to completely cover the silicon. The drop cast samples were subsequently heated at the recorded temperatures for 10 minutes, after which they were rinsed in deionised water to remove vitamin C from the surface.

XPS spectra were acquired using an Axis-Ultra XPS instrument (Kratos Analytical, Manchester, UK) with monochromatic Al Kα X-rays (15 kV, 5 mA, *hν* = 1486.7 eV) in hybrid lens mode. Survey scans (two sweeps) were acquired with a pass energy of 160 eV, step size 1000 meV, and a dwell time of 200 ms. Narrow scans were performed for the C 1s, O 1s, S 2p, and Si 2p regions with a pass energy of 40 eV, step size 100 meV, and a dwell time of 500 ms. Two sweeps were acquired for each of the narrow scan regions. Charge neutralisation, using an electron flood gun, was employed to reduce charging of the samples during measurement. XPS spectra were analysed using CasaXPS software (Version 2.3.16) and intensities were calibrated with the transmission function and average matrix relative sensitivity factors published by the National Physical Laboratory.^40^ Tougaard backgrounds were used for peak quantification from survey spectra except for a minority of cases where a linear background was used instead. Shirley type backgrounds were used for peak fitting of the C 1s narrow scan regions.

## Results and discussion

### Thermal reduction

**Fig. 1:**
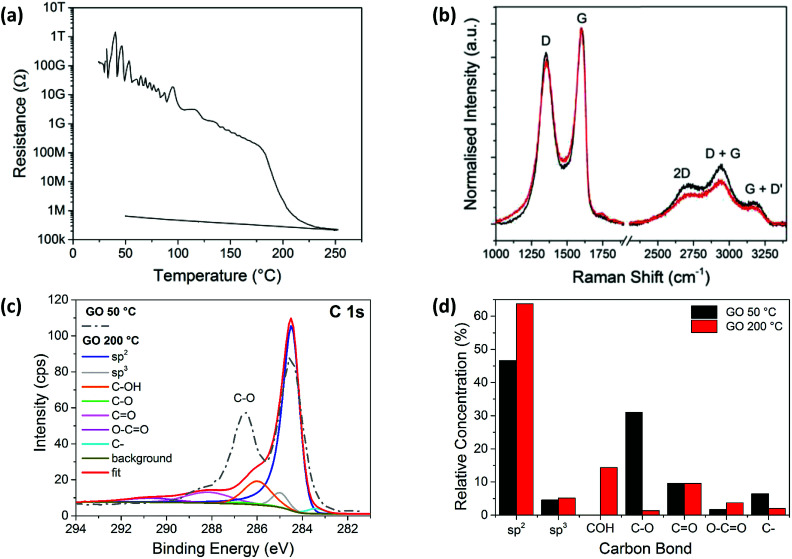
(a) The reduction in electrical resistance of a spray coated GO film on a glass substrate, with temperature increasing from room temperature up to 250 °C and then cooling to 50 °C. (b) Raman spectra of spray coated GO films after heating to 50 °C (black line) and 200 °C (red line), normalised to the G-peak intensity. (c) The XPS C1s spectra corresponding to carbon bonding after heating the GO film to 50 °C (dashed grey line) and 200 °C (red line), with the fitted XPS peaks also shown for 200 °C. (d) Comparison of fitted XPS C1s peaks for samples heated to 50 °C and 200 °C.

Glass substrates coated with GO were heated on a hot plate and the electrical resistance of the films measured to study the thermal reduction of the spray coated GO films in real time. Fig. 1a shows a plot of the GO film resistance when heated to a maximum of 250 °C. The temperature increases at a rate of 40 °C min^−1^ and reaches 250 °C in around 6 minutes. Initially, the GO film is electrically insulating, with a resistance >100 GΩ, but upon heating, the thermally reduced GO becomes electrically conducting with a resistance of ∼200 kΩ. The resistance of the rGO film initially decreases steadily, before decreasing more dramatically as the temperature exceeds 180 °C after 4 minutes, shortly after which the minimum resistance approaches 200 kΩ. The sample is left to cool from 6 minutes onwards, which results in an increase in the resistance to 1 MΩ.

The Raman spectra measured for the films heated at 50 °C and 200 °C, before and after the change in resistance (Fig. 1b), are similar, with *I*_D_/*I*_G(app)_ values of ∼0.85 indicating no significant change in the size of sp^2^ domains, which is consistent with literature.^36,41–43^ This is most likely due to an increase in the number of sp^2^ sites rather than an increase in the sp^2^ domain sizes.^44^ These additional sp^2^ sites form interconnecting pathways which lower the resistance, explaining the changes seen in Fig. 1a.

The XPS spectrum for the GO sample deposited on silicon and heated at 50 °C for 10 minutes, as well as the fitted spectrum for the thermally reduced GO at 200 °C, are shown in Fig. 1c. The relative concentrations of the peak fitted components from both spectra are shown in Fig. 1d. At 50 °C the sp^2^ carbon peak dominates, with a relative concentration of ∼47%, followed by the C–O bond with a relative concentration of ∼32%. However, the C–O bond component decreases to ∼1% for the sample heated at 200 °C. This is accompanied by a marked increase in the sp^2^ carbon peak which is seen to dominate the 200 °C sample spectrum at ∼63%. This clearly indicates a loss of oxygen and suggests that a structure closer to that of pristine graphene is formed.^45^

### Chemical reduction

The oxidation of vitamin C (Fig. S2†) occurs simultaneously with the reduction of GO, resulting in the suspected reaction products of rGO, dehydroascorbic acid and water.^33^ The reaction for the oxidation of vitamin C is shown in the ESI (Fig. S2†) and shows that there is an intermediate stage of the reaction, when only one H^+^ and e^−^ has been lost. At this point the vitamin C is very unstable, quickly leading to further reduction of the GO.

**Fig. 2:**
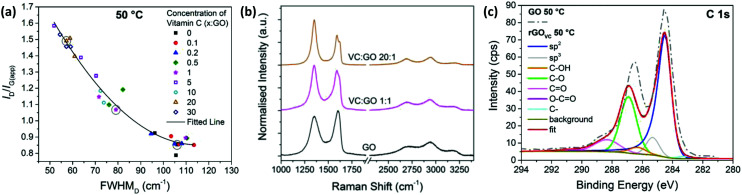
(a) *I*_D_/*I*_G(app)_*versus* FWHM_D_ for Raman spectra from 3 areas on each GO film with different concentrations of vitamin C present, heated at 50 °C for 10 minutes to remove any remaining solvent. (b) Examples of Raman spectra with VC : GO ratios of 20 : 1, 1 : 1 and no vitamin C, corresponding to the circled data points in (a). (c) XPS C1s spectra and fitted peaks for rGO_VC_ at a ratio of 10 : 1 VC : GO, after heating to 50 °C for 10 minutes to remove solvent and a subsequent washing step to remove vitamin C. The XPS C1s spectra of the equivalent GO sample without vitamin C (from Fig. 1c) is also displayed as a dashed line.

Chemical reduction of GO using vitamin C was undertaken as a spray coated film, heated at 50 °C for 10 minutes to remove any remaining solvent and characterised with Raman spectroscopy (Fig. 2a and b). The level of disorder for a graphitic material can be determined from analysis of the Raman G- and D-peaks, using the peak intensity ratio (*I*_D_/*I*_G(app)_) and the full width at half maximum of the D-peak (FWHM_D_).^43,46,47^ Since GO is highly disordered, an increase in *I*_D_/*I*_G(app)_ indicates an increase in sp^2^ rings and therefore ordering, as does the narrowing of the D-peak.^43^ This is consistent with the relationship found in amorphous carbon thin films.^48^

Measured after the chemical reduction process, *I*_D_/*I*_G(app)_ increases with increasing concentration of vitamin C, while the FWHM_D_ decreases with increasing vitamin C concentration. Therefore, these two relationships show there is a lower level of disorder in the GO flakes after chemical reduction with higher concentrations of vitamin C present, indicating more complete reduction. Unlike Fig. 1b, the sp^2^ domain sizes have likely increased, as well as the number of sp^2^ sites. The higher the concentration of vitamin C, the more vitamin C available to react with the oxygen sites on the GO.

From Fig. 2a, for vitamin C : GO (VC : GO) ratios of 0.5–10, the data shows a large variation in *I*_D_/*I*_G(app)_ and FWHM_D_ across one substrate, as shown by the three data points for 3 different spectra at each concentration. This could be due to some flakes being reduced by the presence of vitamin C, while others are not due to the limited supply of vitamin C. Fig. 2b visually demonstrates the changes in Raman spectra between a highly reduced GO film with a 20 : 1 VC : GO ratio, a slightly reduced GO film with a 1 : 1 VC : GO ratio and GO itself (*i.e.* no reduction step). The data points corresponding to the analysed spectra from Fig. 2b are circled in Fig. 2a.

The XPS spectra in Fig. 2c, for the rGO_VC_ reduced at 50 °C for 10 minutes, is directly compared to the GO without vitamin C prepared at the same temperature (from Fig. 1c). The XPS spectra are similar, however the carbon/oxygen (C/O) content increases from 2.1 (GO) to 3 (rGO_VC_, 50 °C), as calculated from the survey spectra.

The Raman spectroscopy and XPS data shown in Fig. 2 indicates an increased reduction of the GO correlated with an increase in vitamin C concentration. The Raman spectra also indicates a lower level of defects for the rGO_VC_ than the electrically conducting GO treated thermally at 200 °C (Fig. 1). However, the effects on resistance before heating are minimal (ESI, Fig. S9a†) and to achieve a noticeable difference without heating, the dispersion has to be left for at least 48 hours before deposition, which also leads to agglomeration of the GO (ESI, Fig. S3†) leaving it difficult to deposit as a thin film *via* spray coating. In industrial applications, shorter timeframes are desired which cannot be achieved by thermal or chemical (vitamin C) reduction alone, therefore a combined approach utilising simultaneous chemical and thermal reduction is examined to attempt to achieve a rapid reduction solution.

### Simultaneous thermal and chemical reduction

The combination of chemical and thermal reduction allows for the rapid realisation of an electrically conducting thin film. Most importantly, vitamin C allows reduction at lower temperatures, while still resulting in a higher conductivity than GO that was thermally reduced at 200 °C.

**Fig. 3:**
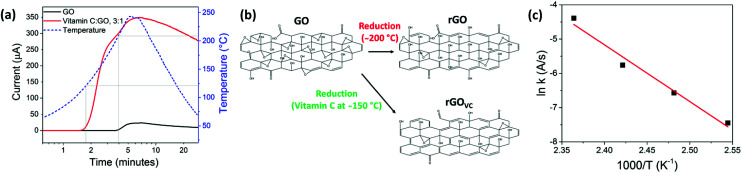
(a) The effect of heating on the electrical current through spray coated GO (black line), and rGO_VC_ (red line) films, the latter deposited from a 3 mg ml^−1^ vitamin C solution. The temperature of the film during heating is also shown (blue dashed line), grey lines indicate the temperature at which the current increases. (b) Chemical structure of GO before and after reduction at 200 °C and with vitamin C at 150 °C. (c) Arrhenius plot for rGO_VC_ at a ratio of 5 : 1 VC : GO and heated at constant temperatures of 120, 130, 140 and 150 °C.

The results in Fig. 3a were obtained in real time by continually recording the electrical current through an rGO_VC_ film, at a constant voltage of 5 V, while the temperature was increased from 25 °C to 250 °C. Unlike in the Raman spectroscopy and XPS measurements in Fig. 2 that reveal changes in the material, the electrical current shows negligible change when heated up to 100 °C in the presence of vitamin C (Fig. 3a). However, when heating above 100 °C in the presence of vitamin C, the current increases significantly and at a lower temperature (120–160 °C) than when vitamin C is absent (210–230 °C). The resistance of the rGO_VC_ sample decreases in two stages, which can be determined from Fig. 3a. Firstly, at 115–165 °C there is an increase in the flow of current, which leads to the conclusion that the rapid reduction reaction between the vitamin C and the GO initiates at ∼115 °C. After 175 °C the rate of current increase slows. Then, at around 200 °C the current in the rGO_VC_ film increases again with the additional thermal reduction of GO, as shown in both Fig. 1a and 3a, for GO without vitamin C. Fig. 3b demonstrates the increase in reduction of oxygen functional groups when vitamin C is added and heated to 150 °C, compared to reduction at 200 °C without vitamin C.

Vitamin C oxidises over time when exposed to air and the oxidation rate increases with temperature,^49^ until it decomposes at ∼200 °C.^50^ The oxidation of vitamin C (ESI, Fig. S2†) facilitates the reduction of GO shown in Fig. 3b (which also increases with temperature), resulting in higher reduction rates at lower temperatures. The reaction rate at different temperatures (ESI, Fig. S4†) can be used to create an Arrhenius plot (Fig. 3c) where the rate constant (*k*) is directly related to temperature (*T*),

1 *k* = *A*e^−*E*_a_/*RT*^

where *A* is a pre-exponential factor, *E*_a_ is the activation energy and *R* the gas constant. Using the Arrhenius eqn (1),^51^ the slope of the line can be equated to −*E*_a_/*R*, giving *E*_a_ = 138 kJ mol^−1^. This is competitive with hydrazine and other alternative methods of reduction.^52–54^ The uncertainty in the measurements was found to be significantly low, as revealed by the error bars in Fig. 3c, which are too small to be observed.

The initial electrical resistance can be affected by the surface coverage, depending on the amount of GO deposited, as demonstrated in Section 1.4 of the ESI (Fig. S5†). Scanning electron microscopy (SEM) images of different concentrations of GO deposited onto silicon substrates are also shown in the ESI (Fig. S6†). As long as the percolation threshold is exceeded,^55^ conducting pathways will begin to form in the deposited GO film as the vitamin C removes functional groups which limit conduction.

**Fig. 4:**
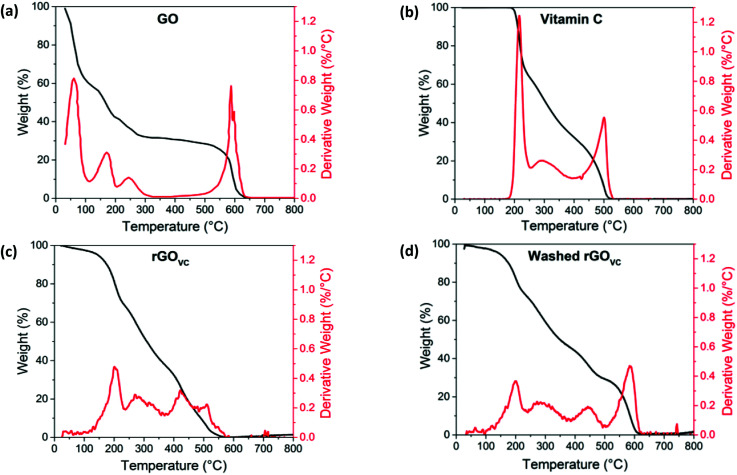
Thermogravimetric analysis in air showing weight (black line) and derivative weight (red line), for (a) GO, (b) vitamin C, and (c) rGO_VC_, at a ratio of 10 : 1 VC : GO after heating at 50 °C and (d) rGO_VC_ as in (c) but washed in DI water before TGA analysis.

Decomposition analysis of GO *via* TGA (Fig. 4a) shows a mass loss step at 50–100 °C caused by evaporation of adsorbed water from the GO surface, two peaks at ∼170 °C and 245 °C due to the thermal reduction of GO^45^ and finally, a peak at ∼590 °C caused by the thermal decomposition of the remaining carbon materials with atmospheric oxygen, in the form of carbon dioxide.^56^

The evaporation of the solvent in Fig. 4a is also observed in Fig. 1a, most likely causing the initial slow decrease in electrical resistance when heating up to 100 °C. The removal of the solvent promotes the formation of more electrical connections between the GO flakes, while also increasing the surface area of the existing electrical junctions. The peak at ∼170 °C, due to the thermal reduction of GO, corresponds with the reduction in resistance above this temperature observed in Fig. 1a, and indicates an increase in conductivity in this material as oxygen groups are removed. The loss of oxygen functional groups at this temperature range is evidenced by the XPS data in Fig. 1c and d, with C–O bonds breaking under the higher thermal energy. GO has also previously been reported to reduce at temperatures of 200 °C.^14,57–59^

For comparison, Fig. 4b shows the TGA results for vitamin C, where two main peaks are observed at ∼215 °C and ∼500 °C, with a gradual loss of material between these two peaks, suggesting that the vitamin C is relatively stable up to 200 °C and then decomposes at higher temperatures. The peak at 500 °C is due to the removal of the remaining amorphous carbon after the vitamin C decomposes.

The thermal decomposition profile of GO with vitamin C (Fig. 4c and d) is effectively a combination of the TGA profiles for GO and vitamin C individually (Fig. 4a and b respectively). Thermal analysis of rGO_VC_ (Fig. 4c) follows a very similar trend to the vitamin C, with only a slight contribution related to the GO decomposition. This is due to the high proportion of vitamin C in the sample (10 : 1). In comparison, the washed sample (Fig. 4d) is a similar combination of the two decompositions seen in Fig. 4a and b, however the vitamin C decomposition is not as prominent due to the washing step removing some of the vitamin C. Gradual weight loss below 150 °C is likely to be due to the evaporation of solvent, but a much smaller weight loss is observed compared to the GO film in Fig. 4a, as in this case no drying step was undertaken before the TGA was performed, unlike for the rGO_VC_ samples.

Fig. 4c and d show the shoulder of the ∼170 °C peak associated with GO reduction. The initial breakdown of the vitamin C (at ∼200 °C) remains, as does the small peak at ∼300 °C, suggesting at temperatures below 200 °C the vitamin C is stable. The peak at 440 °C in both Fig. 4c and d is due to the removal of amorphous carbon left from the decomposition of the vitamin C at lower temperatures, possibly reduced in temperature due to the lower stability after the reaction with GO.^33,60^ In Fig. 4c, the sample has not had a washing step and therefore contains a higher proportion of vitamin C than GO, whereas in Fig. 4d, the sample was washed, leaving a lower percentage of vitamin C, allowing the GO decomposition peak at 600 °C to become more prominent. The derivative weight graphs are overlaid in the ESI (Fig. S7†), in order to demonstrate the overlap of these peaks.

**Fig. 5:**
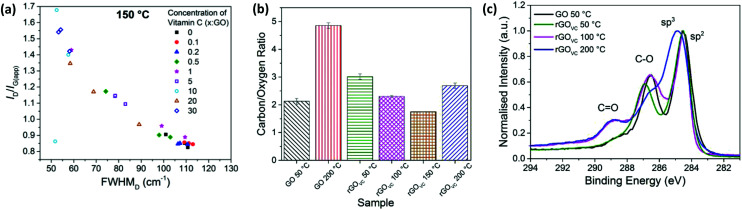
(a) Raman spectroscopy results showing *I*_D_/*I*_G(app)_*vs.* FWHM_D_ for spray coated rGO_VC_ with different concentrations of vitamin C, heated for 10 minutes at 150 °C. Three measurements are recorded for each sample. (b) The carbon/oxygen ratio calculated from XPS survey spectra for GO and rGO_VC_ with VC : GO at 10 : 1, heated at the stated temperatures and subsequently washed. (c) XPS C 1s spectra for rGO_VC_ with VC : GO at 10 : 1, heated at the stated temperatures and subsequently washed, and compared to the spectra for GO heated at 50 °C.

With varied concentrations of vitamin C, GO solutions were spray-coated onto silicon substrates and heated for 10 minutes at 50, 100, 150 or 200 °C. The relationship of the Raman scattering signal with the concentration of vitamin C is similar for the 50 °C, 100 °C and 150 °C samples, with the values for the 150 °C sample shown in Fig. 5a. At temperatures up to 150 °C, the FWHM_D_ reduces, from ∼110 cm^−1^ to a minimum of ∼50 cm^−1^, and the *I*_D_/*I*_G(app)_ ratio increases, from ∼0.8 up to a maximum of ∼1.7, as the vitamin C concentration increases. When heating each sample for 10 minutes, the trends in the level of disorder are more susceptible to changes in vitamin C concentration than they are to changes in temperature (Fig. 2a and 5a).

When the rGO_VC_ films are heated at 200 °C, the *I*_D_/*I*_G(app)_ ratio follows a different trend primarily due to the evolution of Raman peaks caused by the vitamin C present (ESI, Fig. S8a†). At lower temperatures the Raman spectra of the vitamin C does not overlap with that of the GO, however when the vitamin C starts to decompose at around 200 °C, a high background signal combined with a peak at ∼1580 cm^−1^ occurs. For comparison, the Raman spectra for vitamin C at 50 °C and 200 °C, along with the overlap of the GO spectra, can be found in Fig. S8b and c of the ESI.† This decomposition of vitamin C is therefore directly correlated to the differences in the Raman spectra for samples heated at 200 °C, compared with samples heated to lower temperatures.

The difference in the final electrical resistance value (Fig. 3a) after the heating of GO and rGO_VC_ is due to the vitamin C in rGO_VC_ reacting with more of the functional groups on the GO and effectively reducing the level of disorder in GO, as confirmed by the Raman spectra (Fig. 5a). The decrease in the level of disorder would suggest that some functional groups have been removed, which were most likely inhibiting the flow of current. A lower level of disorder is expected to increase the electrical conductivity.^5,44,61^ Even after heating, the decrease in the level of disorder is still apparent for increasing concentrations of vitamin C (Fig. 5). However, the sheet resistance does not improve with increasing concentrations due to the additional vitamin C present (ESI, Fig. S9b†).

When altering the concentration of vitamin C, there is an optimum VC : GO range of between 3 : 1 and 5 : 1. When using too little vitamin C, there is insufficient reduction, as seen in the Raman spectra (Fig. 1b and 5a) and electrical measurements (Fig. 1a). When using too much vitamin C, it coats the flakes and interrupts the electrically conducting pathways, as inferred from the electrical measurements and observed optically, in the ESI Fig. S9 and S10† respectively.

**Fig. 6:**
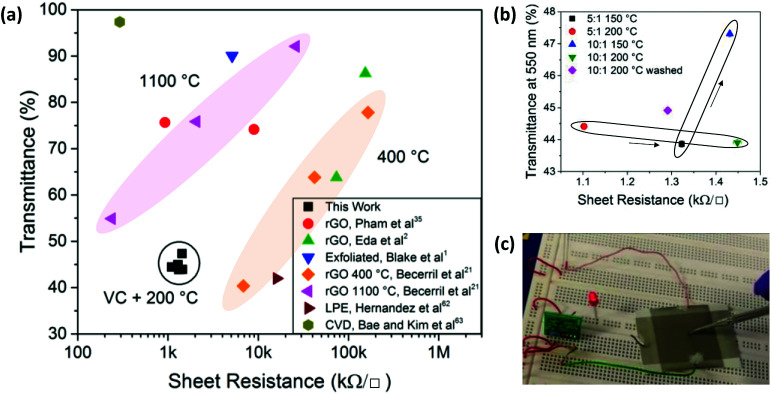
(a) Sheet resistance and optical transmittance for graphene samples comparing rGO data from Pham *et al.*,^35^ Eda *et al.*,^2^ Blake *et al.*,^1^ Becerril *et al.*,^21^ Hernandez *et al.*,^62^ Bae and Kim *et al.*,^63^ and rGO_VC_ in this work. (b) Sheet resistance and optical transmittance for rGO_VC_ on glass using different VC : GO ratios, heating temperatures and washing steps. (c) Operation of a capacitive sensor, activating an LED upon contacting the sensor with an electrically isolated metal probe.

The C/O ratios for GO and 10 : 1 VC : GO heated at different temperatures are shown in Fig. 5b. All the samples were heated for 10 minutes at the stated temperature, after which the rGO_VC_ samples were washed with deionised water to remove residual surface vitamin C before XPS analysis. The error bars in Fig. 6b are too small to be observed, since the uncertainty in the measurements were found to be significantly low. As shown in Fig. 5b, after heating the GO, the C/O ratio increases from ∼2.1 for 50 °C to ∼4.8 for 200 °C. The addition of vitamin C and heating to 50 °C increases the C/O ratio from ∼2.1 (GO) to ∼3 (VC : GO 10 : 1), a reduction in oxygen content due to the vitamin C, as also observed in the XPS C 1s spectra in Fig. 1c and 2c. However, when heating rGO_VC_ up to 100 °C, 150 °C and 200 °C, the C/O ratio decreases relative to the rGO_VC_ 50 °C sample. This is likely to be due to variations in the vitamin C residue in the sample, which varies with the solubility of the vitamin C, oxidised vitamin C and amorphous carbon. To support this, the TGA in Fig. 4d confirms the presence of vitamin C even after a washing process. Furthermore, the Raman spectroscopy results in Fig. 5a confirm that the increase in VC : GO concentration increases the reduction of the GO.

In Fig. 5c, the carbon content is studied in more detail using the XPS C 1s spectra. The proportion of C=O bonds is low for rGO_VC_ at 50 °C, however it increases for 100 °C and 200 °C. This could be due to the presence of the vitamin C, especially if it is present in its oxidised state (ESI, Fig. S2†) when reducing the GO, producing multiple C=O bonds in the place of C–O bonds.^33,60^

### Application

The use of low temperature annealing and spray coating of rGO_VC_ allows for applications utilising temperature sensitive and flexible substrates, such as polymers. When even lower temperatures are required, a significant level of reduction can still be achieved if longer reduction times are used. Fig. 3c shows that the GO can still be reduced at lower temperatures, but at a much slower rate.

Fig. 6a shows the sheet resistance and transmittance for these rGO_VC_ samples relative to other values reported in literature.^1,2,21,35,62,63^ rGO films with sheet resistances that are as low as in this work require higher annealing temperatures^1,21,35^ or reduction with hydrazine.^35^ These rGO_VC_ samples have a lower resistance than rGO reduced at 400 °C, but higher than 1100 °C. Further optimisation may improve the deposition and subsequent transmittance of the rGO_VC_ films as well, to reach values closer to those achieved with high temperature thermal annealing (1100 °C). Higher temperatures such as 400 °C combined with vitamin C may improve the resistance and transmittance further than the reported values at 1100 °C, however the low temperatures are more desirable for industry. From Fig. 6b, the minimum sheet resistance of 1.1 kΩ □^−1^ was recorded for a film of rGO_VC_ with VC : GO of 5 : 1, heated to 200 °C, with a corresponding optical transmittance of 44.4% at a wavelength of 550 nm. When comparing the performances of different VC : GO concentrations, the resistance for a concentration of 5 : 1 heated at 150 °C VC : GO is lower (∼1.32 kΩ □^−1^) than for a concentration of 10 : 1 (∼1.43 kΩ □^−1^ at 150 °C); the latter possibly due to the presence of excess vitamin C. This is highlighted by the inclusion of a washing process, with a 10 : 1 sample after washing producing a sheet resistance of ∼1.3 kΩ □^−1^ compared to ∼1.45 kΩ □^−1^ for a 10 : 1 sample without the washing process (Fig. 6b).

One possible application for the conductive spray coated rGO_VC_ film on a temperature sensitive plastic substrate is a touch sensor, capable of detecting human touch even through a thick insulating barrier, such as a glove, if the circuit components are selected accordingly. A common thermoplastic polymer, acrylonitrile butadiene styrene (ABS), with a glass transition temperature of 105 °C, was used as the substrate for a spray-coated rGO_VC_ layer defining the sensing area. ABS is used for injection moulded and extruded items, such as household and consumer products, enclosures for electrical and electronic assemblies, luggage and protective carrying cases, and automotive trim, amongst others, and has also recently found use in 3D printing.^64–66^

The rGO_VC_ layer on ABS was thermally annealed in an oven for 48 hours at 80 °C (sufficiently lower than the polymer glass transition temperature to avoid changes in the polymer) after spray coating. The electronics required to connect the sensor circuit to an indicator LED are provided in the ESI, Fig. S11.† After activating the circuit, any charge disturbance, such as a conducting object touching the surface, switches the Integrated Circuit (IC) output terminal and activates the LED attached to the output pin of the IC, as shown in Fig. 6c, demonstrating the suitability of the rGO_VC_ spray coated films for real-world electronics applications.

Shorter annealing time periods can be achieved, which may be more desirable for industrial scale up, when the substrates used can withstand temperatures above 115 °C; one such example polymer being Kapton®, which has historically been used in flexible printed electronics.^67,68^

During chemical and thermal reduction, after heating past the point of rapid reduction (around 150 °C), the junction resistance becomes a key factor (Fig. 6b and S5†). To minimise this, several factors can be optimised. Spraying immediately after adding vitamin C limits agglomeration and produces a more homogeneous rGO thin film. A minimum amount of vitamin C should be used to prevent it from inhibiting the electrical connections between GO flakes, unless the vitamin C is removed by washing afterwards. However, washing can remove GO flakes at the same time, therefore requiring several depositions.

## Conclusions

A combination of chemical and thermal reduction has been used to increase the electrical conductivity of spray coated rGO thin films. This approach decreases both the temperature (<150 °C) and time required (<10 minutes) to reduce the GO thin film into an electrically conducting film, using spray coating deposition techniques. We have shown that the technique can be used to produce conducting rGO films on a thermoplastic polymer, acrylonitrile butadiene styrene (ABS), which is commonly used in industry due to its light weight, but which has an operating temperature of only 80 °C.

The low temperature production methodology has a number of advantages including: compatibility with a variety of temperature sensitive substrate materials; usage of an environmentally-friendly solvent in atmospheric conditions, minimising safety risks; and deposition compatible with a variety of printing and spray coating techniques. With optimisation, deposition of the thin film *via* spray coating creates a relatively homogeneous rGO thin film, minimising the thickness of the material needed to create an electrically conducting semi-transparent film making them suitable for applications such as active packaging, sensor systems, touchscreens and interactive surfaces.

## Conflicts of interest

There are no conflicts to declare.

## Supplementary Material

RA-008-C8RA08849G-s001
