# Supplementary material for: Physicochemical characterisation of reduced graphene oxide for conductive thin films
Source: RSC Adv. 2018 Nov 7;8(65):37540–9. doi: 10.1039/c8ra08849g (PMC9089401; doi:10.1039/c8ra08849g)
Supplement: RA-008-C8RA08849G-s001 [file RA-008-C8RA08849G-s001.pdf]

Electronic Supplementary Material (ESI) for RSC Advances.
This journal is © The Royal Society of Chemistry 2018

Supplementary Information for: Physicochemical Characterisation of Reduced Graphene Oxide for Conductive Thin Films

Lateral Flake size

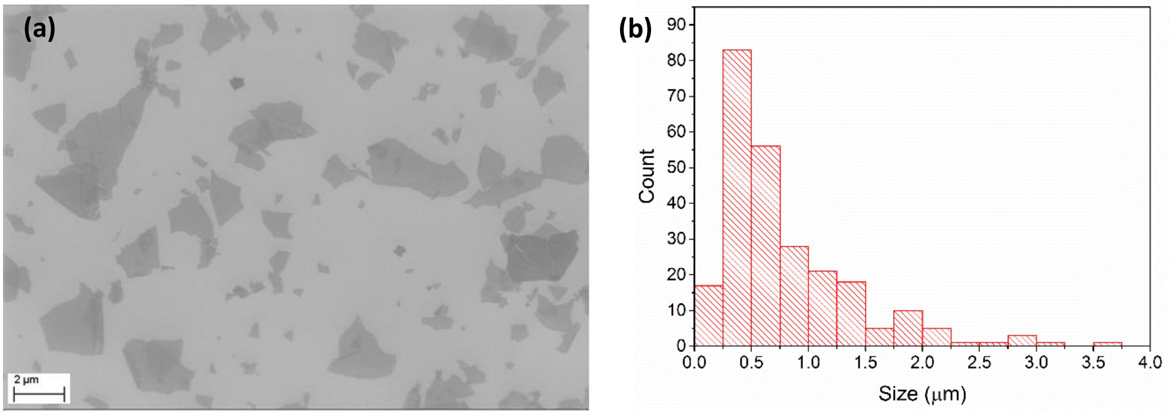

Figure S1. (a) Scanning electron microscope (SEM) image of drop cast graphene oxide flakes (b) Histogram of average flake size.

SEM measurements were performed using a Zeiss (Oberkochen, Germany) Supra Microscope (In lens, 30 µm aperture, 2 kV accelerating voltage).

Modified Hummers and Offeman method

Graphite oxide is produced from graphite as a product of the Hummers and Offeman method.[1] This involves binding oxygen functional groups[2] to the graphite sheets and weakening the van der Waals forces between the graphene layers. Subsequently, when dispersing the graphite oxide in water, sonication produces a high yield of graphene oxide (GO) flakes.

The Hummers and Offeman method uses sulphuric acid (H_2SO_4), sodium nitrate ($NaNO_3$), potassium permanganate ($KMnO_4$), deionised water (H_2O), and hydrogen peroxide (H_2O_2): the sulphuric acid, sodium nitrate and potassium permanganate are the oxidising agents.[3]

Deionised water is used to dilute the solution and reduce the pH to neutral,[4] which terminates the reaction. Hydrogen peroxide is used to reduce the excess potassium permanganate at the end of the reaction.[4]

Vitamin C oxidation and GO reduction

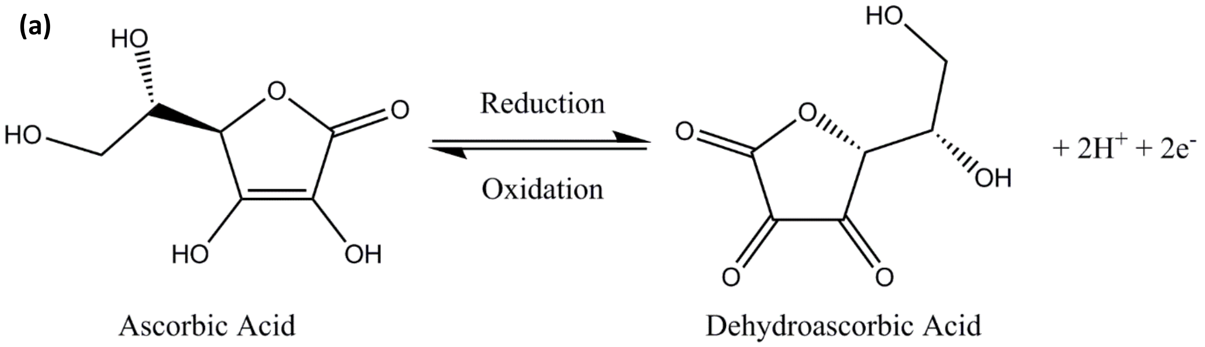

Figure S2. Chemical structure of vitamin C (ascorbic acid) during oxidation and reduction.

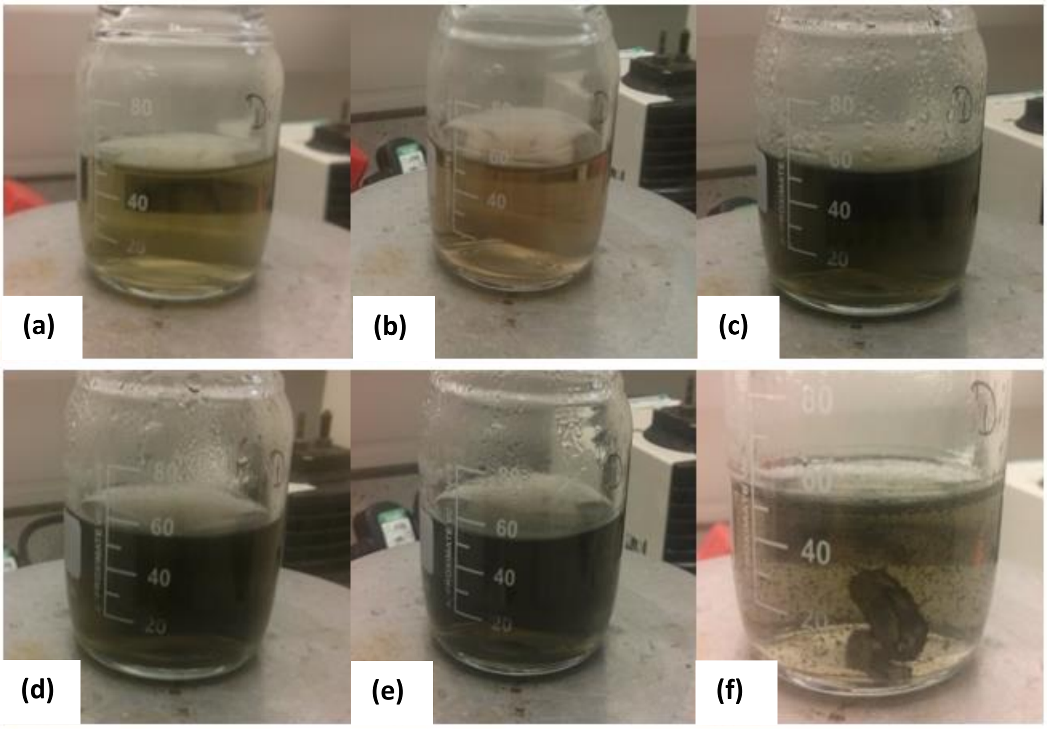

Figure S3. Photographs showing progression of vitamin C reduction of GO, after (a-f) 0, 1, 24, 48, 72, and 144 hours.

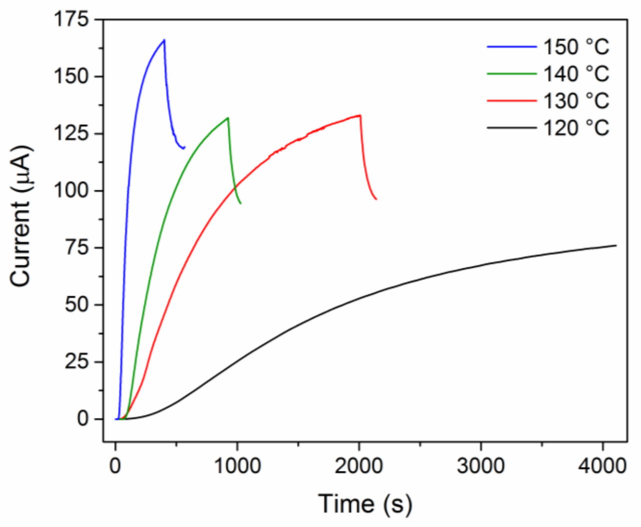

Figure S4. The rate of change of current versus temperature (120-150 °C) for spray coated rGO_{VC} at a ratio of 5:1 for VC:GO. The heating is switched off at the maximum point of each curve.

When comparing temperatures close to the GO insulator transition temperature, Figure S4 demonstrates the effect temperature has on the time taken for the electrical conductivity to change, as well as the rate of change. Here, the higher the temperature, the faster the current increases and the higher the resulting conductivity.

Percolation theory

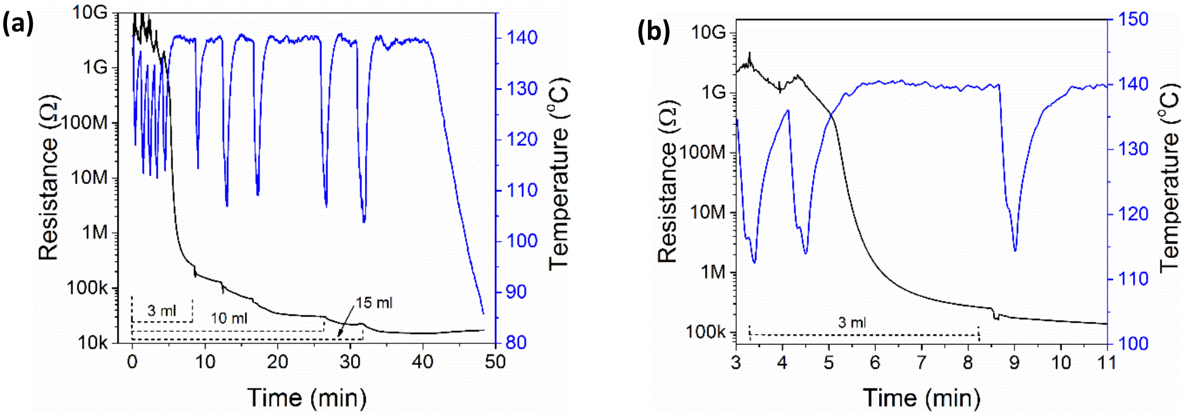

Figure S5. (a) Electrical resistance (black line) dependence on the volume of rGO material deposited onto a pre-heated substrate at 140 °C. Sequential spray coating depositions onto the substrate (indicated by a simultaneous drop in temperature, blue line) are undertaken up to 15 ml of GO solution deposited. (b) A magnified area of (a), showing the major electrical resistance decrease with the deposition of 3 ml of GO solution.

In Figure S5a, the electrical resistance dependence on volume of GO solution deposited is presented. A solution of vitamin C and GO (rGO_{VC}) was periodically spray coated onto a pre-heated (140 °C) glass substrate, with labels on the graph indicating the points at which the total volume sprayed is equal to 3 ml (170 kΩ), 10 ml (30 kΩ) and 15 ml (22 kΩ). The time taken for each deposition is noticeable in the figure as a reduction in temperature, with the subsequent reduction and recovery of the temperature due to the lower initial temperature of the sprayed material and the consequent solvent evaporation. The difference in volume of GO solution deposited, and therefore the number of connecting pathways produced in the deposited GO film after reduction, determines the final electrical resistance. Figure S5(b) is a magnified area of Figure S5(a), which focuses on the point at which a robust percolation pathway occurs, using a volume of only 3ml of VC:GO solution. Further deposition of GO material and subsequent annealing causes the resistance to decrease further, however this also decreases the optical transmittance.[5]

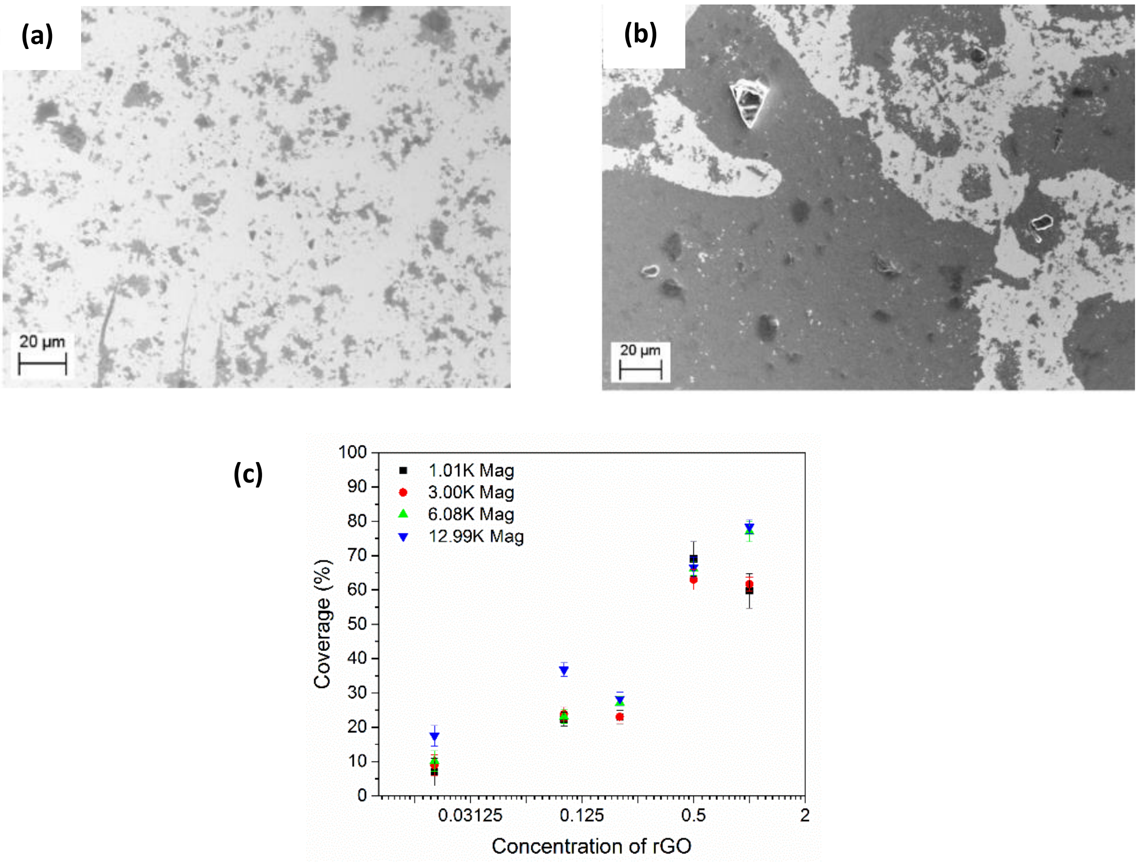

Figure S6. SEM (10 kV accelerating voltage) images of different concentrations of GO deposited onto silicon, increasing from (a) 0.2 mg/ml to (b) 0.5 mg/ml. The GO flakes appear dark. (c) The % coverage depending on the concentration of GO deposited, determined after analysing SEM images at different magnifications in ImageJ.

TGA

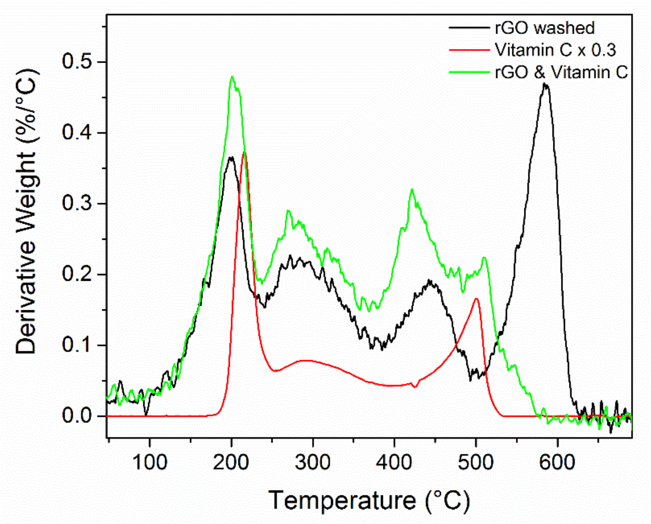

Figure S7. Thermogravimetric analysis showing the derivative weight for vitamin C and rGO_{VC} with and without washing.

Raman Spectroscopy

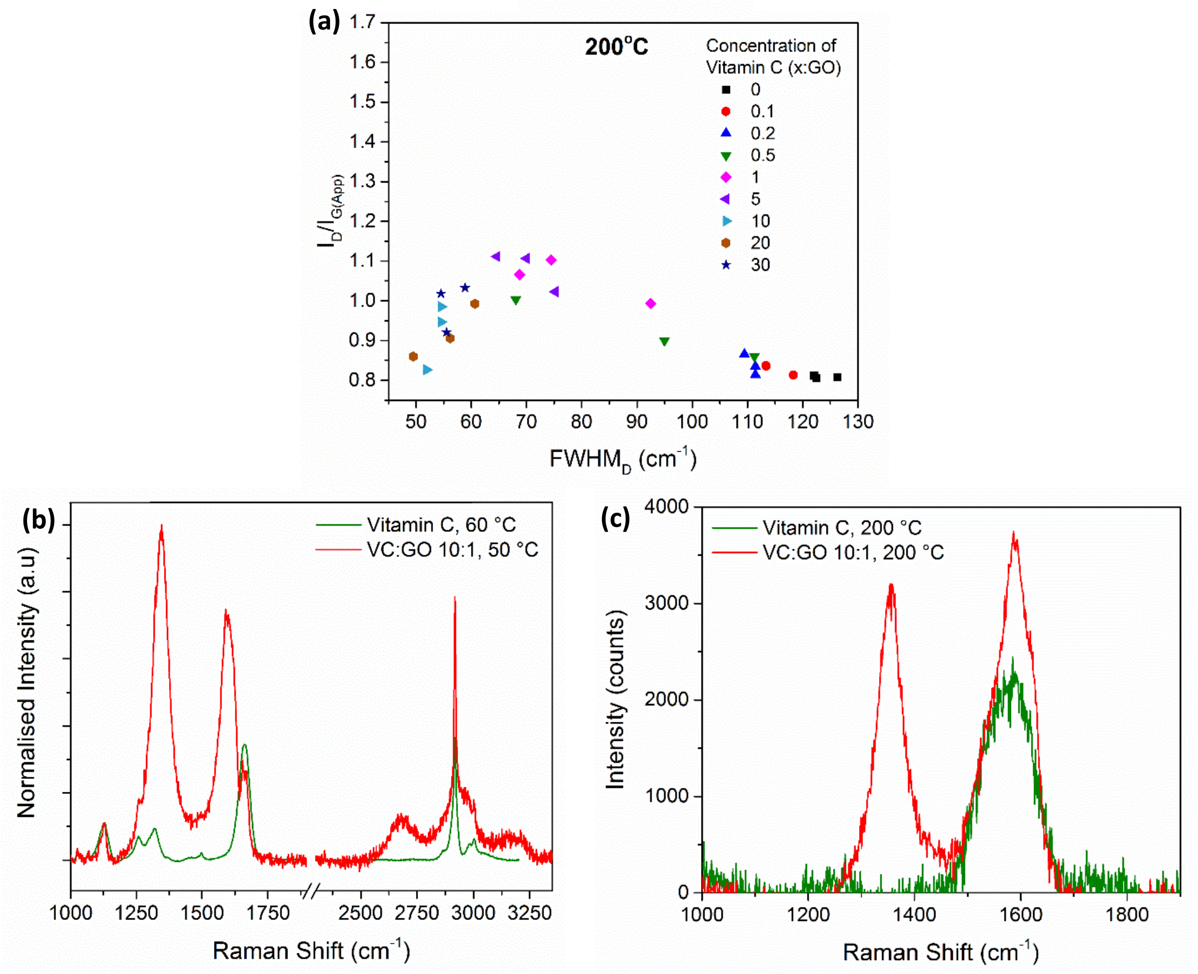

Figure S8. (a) Raman spectroscopy results showing $I_D/I_{G(app)}$ vs $FWHM_D$ for spray coated rGO_{VC} with different concentrations of vitamin C, heated for 10 minutes at 200 °C, 3 measurements are recorded for each sample. (b) Raman spectra of rGO_{VC} with a VC:GO ratio of 10:1, heated at 50 °C, compared with the vitamin C Raman spectra heated at 60 °C. Both spectra are normalised to the peak at 1130 cm^{-1}. (c) A comparison of the Raman spectra of vitamin C and rGO_{VC} with a VC:GO ratio of 10:1, both annealed at 200 °C. The Raman spectra are shown after background subtraction. The vitamin C spectra was taken with a 532 nm laser and all others with a 514 nm laser.

For films annealed at 200 °C, the Raman spectra in Figure S8a shows that with increasing vitamin C concentration $I_D/I_{G(app)}$ again increases from ~0.8, but reaches a maximum at ~1.1, where the $FWHM_D$ is ~70 cm^{-1}, after which the $I_D/I_{G(app)}$ ratio decreases to ~0.8, unlike the films annealed at lower temperatures (Figure 5). Despite the difference in the $I_D/I_{G(app)}$ ratio however, the $FWHM_D$ follows a similar trend for both the 200 °C anneal and the lower

temperature anneals, reaching a minimum of ~50 cm^{-1} at the highest concentrations of vitamin C.

Figure S8b and S8c show the vitamin C Raman signal after heating to 60 °C (to remove any water) and heating to 200 °C. The high vitamin C concentration of 10:1 in Figure S8b, shows the superposition of the vitamin C Raman signal on the rGO spectrum. The noise level in Figure S8c is due to the high background which is seen in the Raman spectra when heating vitamin C.

Reduction with Vitamin C over time

When leaving vitamin C within the solution for a prolonged period of time (48 hours), prior to thermal reduction, the initial resistance of the spray coated film (before any thermal reduction is initiated) is lower than that of a film spray coated after 0 hours by 2 to 3 orders of magnitude, however the resistance after heating is around 1 order of magnitude higher than the solution left with vitamin C for 0 hours (Figure S9a). The temperature at which the resistance initially decreases is similar (105-125 °C), irrespective of how long the GO remains in the vitamin C solution. Optimisation of the output current with vitamin C concentration is shown in Figure S10b.

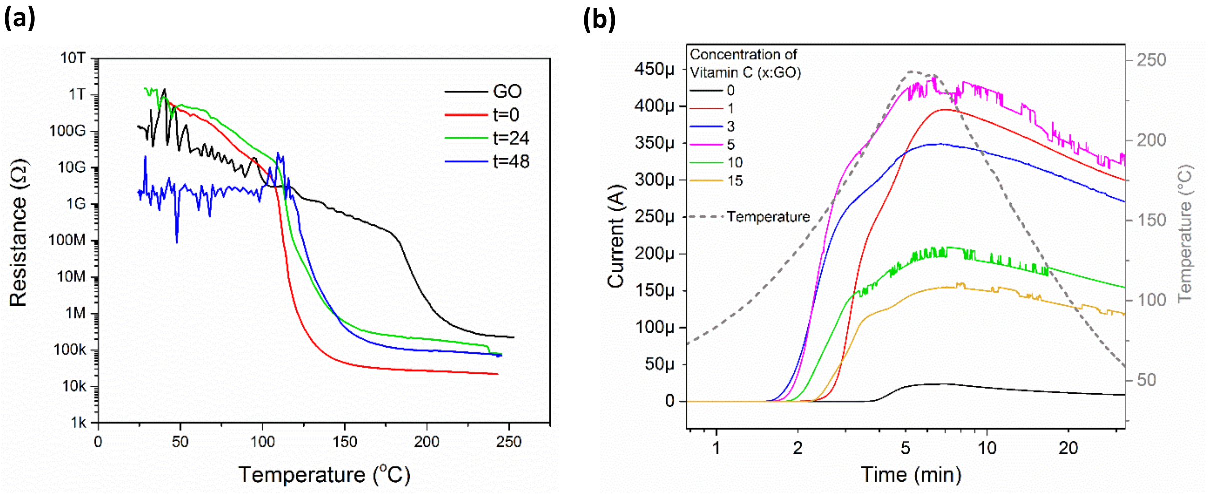

Figure S9. (a) Spray coated GO (black line), and rGO_{VC} spray coated and measured 0, 24, and 48 hours after the inclusion of vitamin C reducing agent (5 mg/mL). (b) The effect of heating on the output electrical current for spray coated GO (black line), and rGO_{VC} films deposited from 1, 3, 5, 10 and 15 mg/mL vitamin C solution

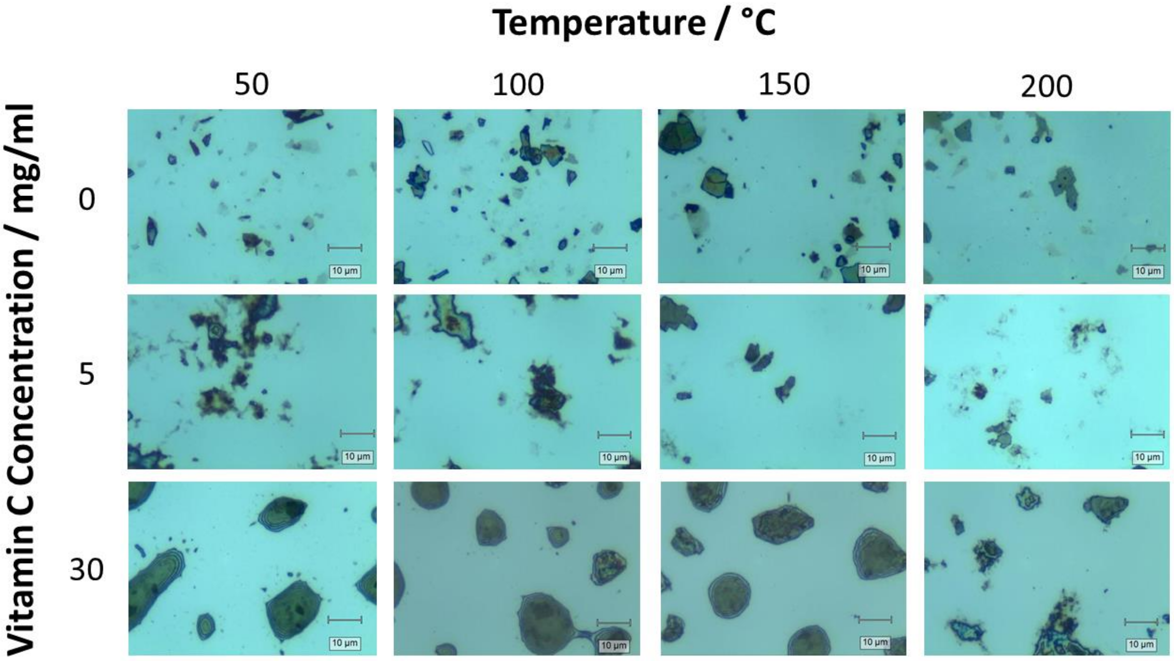

Figure S10. Optical images of GO and rGO_{VC} flakes at 5:1 and 30:1 VC:GO concentration ratios annealed at different temperatures, as stated.

Detector electronics

In order to operate the capacitive sensor produced using the rGO films, an Atmel AT42QT1010 IC was used (Figure S11a). The IC is a digital burst mode charge-transfer capacitive touch sensor controller which allows the device to detect a touch or proximity field. Charge measurement hardware functions and switches are all internal in the IC.

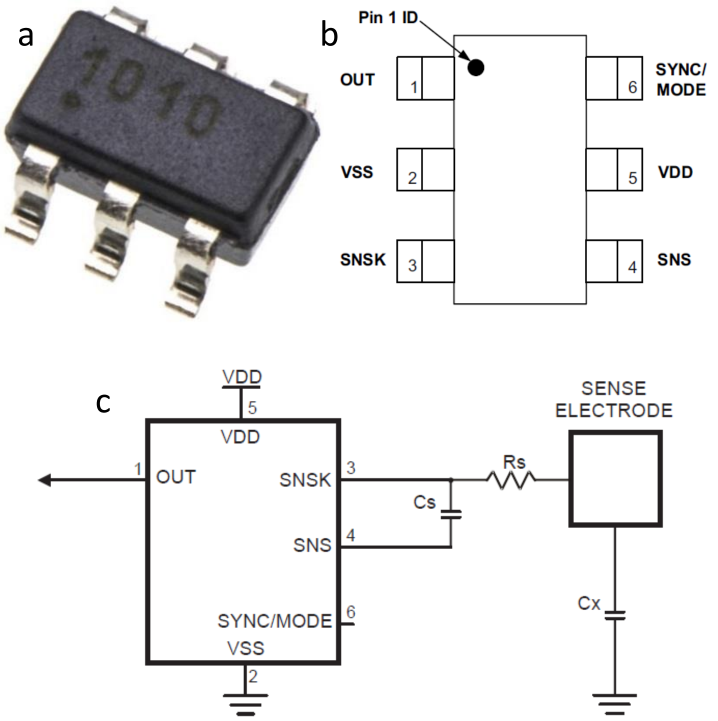

Figure S11. (a) Atmel AT42QT1010 IC. (b) Pinout configuration, and (c) circuit diagram of the SOT23-6 package type IC as per the manufacturer's specification.

References

1 W. S. Hummers and R. E. Offeman, *J. Am. Chem. Soc.*, 1958, **80**, 1339.

2 T. Szabó, O. Berkesi, P. Forgó, K. Josepovits, Y. Sanakis, D. Petridis and I. Dékány, *Chem. Mater.*, 2006, **18**, 2740–2749.

3 A. M. Dimiev and J. M. Tour, *ACS Nano*, 2014, **8**, 3060–3068.

4 M. D. P. Lavin-Lopez, A. Romero, J. Garrido, L. Sanchez-Silva and J. L. Valverde, *Ind. Eng. Chem. Res.*, 2016, **55**, 12836–12847.

5 V. H. Pham, T. V. Cuong, S. H. Hur, E. W. Shin, J. S. Kim, J. S. Chung and E. J. Kim, *Carbon N. Y.*, 2010, **48**, 1945–1951.
